# An Exploratory Study of the Social Contract of Research and Innovation in Africa

**DOI:** 10.3389/frma.2022.849263

**Published:** 2022-06-09

**Authors:** Michael Jeffrey Kahn

**Affiliations:** Centre for Research on Evaluation, Science and Technology, DSI-NRF Centre of Excellence in Scientometrics and STI Policy, Faculty of Arts and Social Sciences, Stellenbosch University, Stellenbosch, South Africa

**Keywords:** social contract, innovation policy, industrial policy, underdevelopment, dependency theory, theory of change, innovation systems

## Abstract

This exploratory study adds to the under-developed literature on a Research Topic that laden with epistemological, philosophical, and ideological overtones, and that begs many questions. The literature on political economy generally, and that for Africa, enjoys full disciplinary status. In contrast the political economy of research and innovation remains an emerging interdisciplinary field that examines the overlap between innovation studies and political economy. The pursuit of “science and technology” was expected to play its part in the imperialist and colonial agendas, and in the post-colonial project, when science and technology policy was a strong element in advocacy for Africa's post-independence development. What have the policies achieved, and what explains the shortfalls? What indeed is the relationship between industrial policy and research and innovation policy? What is the social contract with research and innovation? The study commences with a general overview of the social contract for science before turning to Africa's post-independence modernizing agenda, and the roles ascribed to industrial policy and research and innovation policy. An eclectic methodology drawing on Cloutier (2021) is deployed to characterize and measure the social contract between research and innovation. The methodology adapts Cloutier (2021) to the functionality of national innovation systems. The responsiveness of STI policy is further probed using Martin, 2015 categorization of innovation policy informed by Theory of Change. Where possible reference is made to conventional STI indicators. Research and innovation policy is then assessed at continental and national levels, with attention given to the extent of linkages in national innovation systems. Further to tease out the various forms of social contract, five country-level STI policies are analyzed using the Martin categorization and Theory of Change methodology. It will be argued that a binding, social contract for inclusive research and innovation policy is largely absent, so that the prospects for attaining the SDGs remain elusive. Post Glasgow COP-26, donor pressure might be re-oriented to promote engagement with the Sustainable Development Goals, though the upholding research sovereignty could mitigate against this. Africa might rightly chide against such pressure, given her experience of what has been labeled as “vaccine apartheid.”

## Introduction

This Research Topic investigates the relationship between political economy and research, STI, and knowledge systems in Africa through the lens of the social contract. By its very nature the investigation is exceptionally broad, as it must cover political economy, history, sociology, and anthropology in the effort to situate “research and innovation” policy and to link this with national and continental development agendas.

This contribution adds to the under-developed literature on a topic laden with epistemological, philosophical, and ideological overtones, and that begs many questions.

Political economy is understood as the interaction among society, politics, culture, and the economy. Innovation studies emerged in the advanced and recovering economies in the post-war period and spread out across the globe in the quest to understand learning, innovation, and competence building.^1^ A new urgency attaches to the expectation of innovation studies, to enable attainment of the Sustainable Development Goals, and most pressingly to understand and mitigate the COVID-19 Pandemic and future zoonotic disease outbreaks.

While the literature on African political economy, historical, anthropological, and sociological studies enjoy full disciplinary status, the political economy of research and innovation is an emerging, interdisciplinary field that examines the overlap between innovation studies and political economy. The pursuit of “science and technology” was expected to play its part in the imperialist and colonial agendas, and in the post-colonial project, when science and technology policy was a strong element in advocacy for Africa's post-independence development. Meeting the Sustainable Development Goals (SDGs), in these in the shadows of the Anthropocene and COVID-19 Pandemic forces an appraisal. What have the policies achieved, and what explains the shortfalls? What indeed is the relationship between industrial policy and research and innovation policy? What is the social contract with research and innovation?

The study commences with a general overview of the social contract for science. It provides an overview of Africa's post-independence modernizing agenda, the role ascribed to industrial policy on one hand, and science and technology policy on the other.

The conceptual frame that will be used to provide tentative answers to these questions is that of the social contract of science, a.k.a. research and innovation, that with few African country exceptions, is a neglected topic. An eclectic methodology adapts Cloutier (2021) measurement of the political social contract to examining the social contract for research and innovation. The methodology probes the functionality of national innovation systems, with specific attention to STI policy instruments using Martin, 2015 categorization informed by Theory of Change. Attention is given to the linkages in national innovation systems, especially between the universities and other research and innovation actors. Five country-level STI policies are analyzed using theory of change methodology. Where possible reference is made to conventional STI indicators.

It is argued that an explicit social contract for inclusive research and innovation policy is largely absent, so that the prospects for attaining the SDGs remain elusive. Post Glasgow COP-26, donor pressure might be re-oriented to promote engagement with the STI interventions needed to attain the Sustainable Development Goals though the national quest for research sovereignty could mitigate against this. Africa might rightly chide against such pressure, given her experience of “vaccine apartheid.”

## Materials and Methods

This section offers a brief review of the literature and proposes a methodology for the subsequent analysis of section Continental and Country Perspectives.

The first sub-section examines the Western origins of the notion of a social contract for science as expressed in the growing interest in the relationship between investment in science and productive activity, covering the period from before World War II to the present. The second subsection makes the explicit link between innovation policy and industrial policy, and often neglected area of inquiry. The third subsection proposes a novel way of measuring the existence and character of the social contract for research and innovation (science).

### The Social Contract and Science Policy

The goal of this study is bold, and as Taylor (2006) notes, “no one has yet been able to explain why some countries succeed at S&T while others fail, or why national S&T success tends not to last long.” Why then do some countries succeed in the economic domain while others fail, or decline from prior success? Contemporary explanations of the drivers of national economic success are many, as in Landes (1995) who considers natural endowments, disease, and technological expertise, Fukuyama (2011) whose concept of the “natural state” serves to explain the prevalence of patrimonialism, Acemoglu and Robinson (2012) who add in the institutional dimension, and others such as Dubow (2006) on the historico-social, Tyfield (2012), the cultural, and Taylor and Wilson (2012) who revisit openness. Baldwin (2016) distills economic success down to the ability to absorb and adapt others' science and technology, actions that are predicated on the capacity to learn. Chang et al. (2016), refer to this as productive capability. More recently the World Bank (Independent Evaluation Group, 2019) has drawn attention to the growing interest in applying social contract theory to the problems of development, especially policy implementation failure, the wicked problem of binding constraints, and state resilience to fragility (Cloutier, 2021).

Bernal (1939) provides a convenient point of departure for a discussion of modern approaches to the relationship between science, production, and society, adopting a Marxian approach in examining the social responsiveness of science. In the United States, Bush (1945) drew on wartime experience to argue for government investment in use-oriented basic science. Bernal and Bush provide examples of instrumentalist approaches that in contemporary parlance might be termed their respective theories of change.

Post-war reconstruction and the social and economic development saw emergence of the new discipline of development economics *a la* Hirschman (1958) gaining importance. Hirschman is credited with this new emphasis, providing the intellectual base for the Sussex Manifesto (Singer et al., 1970) to build capability, halt external brain drain, and restrict “internal brain drain.” Here too, the call for socially responsive science. Not to be overlooked is the seminal work of Merton (1973), the founder of the sociology of science, who offered scientists the idea of CUDOS—a set of behavioral norms for science. Soon thereafter came Lord Rothschild's “customer-contractor” principle that set out to ensure the accountability of publicly funded science (HM Government, 1971). In the West a range of forces impacted on the science-society debate. The Campaign for Nuclear Disarmament; the anti-war movement; emerging “value-for-money” governance; nascent environmental campaigning; the Club of Rome (1972) *Limits to Growth*.

An important voice was that of Ravetz (1988) who studied the social embeddedness of science. Latour (1987) later avowed that science and technology could only be studied “in action” and that such action was embedded in network activities. Then, in a highly influential publication, Gibbons (1999) suggested that blue sky discipline-based science, termed “Mode 1,” had given way to a new paradigm of socially distributed, trans-disciplinary, funder-driven, and application-oriented science, designated “Mode 2.” This seeded a conflict between policy makers and civil servants on the one side, and researchers on the other. The former found a convenient fit between Mode 2 and the precepts of New Public Management (Hood, 1995) that was then in vogue; the latter reacted to the imposition of Mode 2 as an assault upon their academic freedom. Mode 2 shows some resonance with the innovation systems formalism that emerged at various sites in the 1980s (Lundvall, 1985; Freeman, 1987). Though lacking a prescriptive edge, the idea of innovation systems is a useful way of understanding the complex, non-linear, multi-actor interactions of the innovation process. Perez (2002) notably examined the interaction between technology (science), economics, society and financial systems captured in the idea of “techno-economic paradigm.” In the United States several influential voices emerged. Lubchenco (1998) pushed for a commitment to pressing societal problems in exchange for public funding. Guston (2000) followed with the riposte that the old contract of self-regulation and linear model thinking should be retired.

These currents fed into the World Conference on Science, whose *Bangalore Declaration* called for a multidisciplinary enterprise of science that would “show a human face” with attention to “inequalities, poverty, social injustice, inadequate health care and education and environmental degradation” (UNESCO, 1999: 2). Next followed the 2002 World Summit on Sustainable Development hosted in Johannesburg by South Africa. In the run-up to the meeting, South Africa declared the need for a new social contract between science and society, capacity-building to narrow knowledge and technology divides, increased inter-disciplinarity, and increased dialogue between scientists and policy makers. Yet the resulting *Johannesburg Declaration* and its associated *Plan of Implementation* made no explicit mention of a contract between science and society (UNESCO, 2002), rather stressing the expert role of scientists and technologists. The world science community reverted to the precepts of the Republic of Science (Polanyi, 1962): we know and advise through our expert channels.

Enter Marburger, science adviser to President Bush, who called for “econometric models that encompass enough variables in a sufficient number of countries to produce reasonable simulations of the effect of specific policy choices (Marburger, 2005: 1087).” Such was the hubris before the 2007 financial implosion. It fell to (Mazzucato, 2011 :23) to offer a critique of action. First in re-emphasizing the importance of networks, and then in reminding that the “lean state” was a fiction: “It is the state as catalyst, and lead investor, sparking the initial reaction in a network that will then cause knowledge to spread.” This shifted the parameters of the social contract to one that blurred the supposed divide between public and private sector science, and hopefully innovation. Mazzucato, 2021 thinking, with its revival of “mission oriented” research and innovation, has garnered widespread interest in both North and South.^2^

Then Elzinga (2012) who provided a threefold periodization: legitimation; professionalization; and lastly accountability, the latter coinciding with the rise of New Public Management. The above debates often miss the ongoing financial crisis and sustainability and climate change. Krishna (2014) for example sees the emergence of a new social contract for science that mimics the market dominance of globalization, amounting to acceptance of business as usual.

Working from the economic perspective Martin (2015) identified a twenty-stage progression of science policy, from early the days of the individual entrepreneur and the role of corporates (Bush, 1945) and Rothschild (1972) (see Table 1 below). Among other contributors cited earlier, Lundvall (1985) maps system thinking and Gibbons (1999) to Mode 1/2, with Perez (2002) on system failure. Some influential economists in the Martin typology would include Arrow (1962)—three factors, Nelson and Winter (1982)—evolutionary economics, Romer (1986)—new growth theory, Cohen and Levinthal (1989)—the two faces of R&D, Malerba (2002)—sectoral systems, Chesbrough (2003) and (Füller et al., 2013)—user innovation. It is the influence of economic analysis and its impact on the shape of innovation policy that persuades for the use of the progression as a reference tool.

**Table 1:** Evolution of science policy.

**1**.	**Individual entrepreneur to corporate innovators**	**11**.	**From neoclassical to evolutionary economics**
**2**.	***Laissez faire* to government intervention**	**12**.	**From neoclassical to new growth theory**
**3**.	**Two factors of production to three**	**13**.	**From the optimizing firm to resource-based view of the firm**
**4**.	**Single division to multidivisional effects**	**14**.	**From individual actors to systems of innovation**
**5**.	**Technology adoption to innovation diffusion**	**15**.	**From market failure to system failure**
**6**.	**Science push to demand pull?**	**16**.	**From one to two “faces” of R&D**
**7**.	**Single to multi-factor explanations of innovation**	**17**.	**From “Mode 1” to “Mode 2”**
**8**.	**From a static to a dynamic model of innovation**	**18**.	**From single technology to multi-technology firms**
**9**.	**From linear model to “chain-link” model**	**19**.	**From national to multi-level systems of innovation**
**10**.	**One innovation process to sector-specific types**	**20**.	**From closed to open innovation**

This sets the stage for the contribution of Schot and Steinmuller (2018), that might be termed Sussex Manifesto redux. They identify two prior frames of innovation policy—innovation through R&D, the innovation systems approach, and a the third that acknowledges unintended consequences, negative externalities, poverty and exclusion, the challenge of the SDGs. This amounts to a bold vision that skirts the issue of political formations and the management of conservative (not conservation) interests. More recently, Chataway et al. (2019) investigated the mechanisms for state funding of science, arguing that innovation systems approach, Mode 2, and demands for greater accountability notwithstanding, Republic of Science thinking holds sway. Chataway et al. (2019) further suggest that Evans (1995) concept of “embedded autonomy” be extended to these debates. His theory received critical review (Wright, 1996) for its lack of empirical precision.

The adoption of the consensus-laden Sustainable Development Goals could imply a shift toward a socially inclusive contract for science, with some progress having emerged through the Glasgow COP-26 November 2021 resolutions.

### Science and Industrial Policy

Following Rosenberg (1974), the next matter is the relationship between science and production as expressed in industrial policy (see, e.g., Mowery and Rosenberg, 1999; Mokyr, 2017).

In response to the crisis of nation building, and inspired, if not induced by the central planning formalisms of the Soviet Union, numerous African governments conceived statist models that offered the semblance of nationhood, holding out the promise of future economic growth. Many governments embarked on programmes of top down industrial policy in nationalizing banks, industry and the commanding heights of their economies, *vide* Angola, Algeria, Egypt, Ethiopia, Ghana, Guinea, Libya, Mozambique, Somalia, Sudan, Tanzania, Zaire, and Zambia.

Economic dependency arising from a lack of control over the terms of trade had exercised the mind of Prebisch (1950), who provided the framework for state-guided import-substitution industrialization (ISI) as the means to mitigate the disadvantages of structural dependency. This gave expression to the early thinking of Dependency Theory wherein the industrializing “metropoles” were cast as dominant over the commodity exporting “periphery.” Baran (1957) and Frank (1966) then added revolutionary appeal to Dependency Theory in calling for the overthrow of the state, failing which under-development would endure unimpeded. Rodney (1972) extended the analysis to Africa, emphasizing the “underdevelopment” inherent in neo-colonialism. In like vein Leys (1975) provided a critical analysis of class relations and the stranglehold of capital in Kenya.

The idea of structural dependency and its constraints duly entered the rhetoric of African independence (Amin, 1977), being given form in the *1963–2002 Lagos Plan of Action for the Economic Development of Africa* (OAU, 1980) that envisaged technocratic, capable governments that would drive industrial development through harnessing Science and Technology.

Chang et al. (2016), in a key report for the UN Economic Commission for Africa, provide an incisive review of the contribution of industrial policy to African development over the last decade and a half. They note that the first decade ushered in the narrative of “Africa Rising,” as a number of states achieved double digit GDP growth based on the commodity exports. However, the failure to achieve “sustainable growth based on the development of the manufacturing sector … is making the continent's long-term prospect worrisome (idem: xi).” They then argue that manufacturing and high-end services are key to such growth, in that there are almost no cases where a route other than manufacturing has succeeded in lifting all boats. They take note of the prior use of tariff and infant industry protection that enabled the core metropoles to forge ahead, kicking away the very ladder that had enabled their leap forward, this in the form of the World Trade Organization accession rules. Furthermore, the dissemination of global value chains (GVCs) would appear to have shrunk the space for active industrial policy. They suggest that a precondition for manufacturing development is building productive capabilities, that can and should be an aim of national development planning. Secondly, there is no industrial policy blueprint, that can be copied from abroad and implemented in the unique local domain. Industrial policy should therefore be informed by other country practices and be designed to function in the local context.

In such an approach, where resources are constrained, industrial policy leads, and innovation policy follows.

### Method

A means of quantifying the existence and character of the social contract is provided by Cloutier (2021) quantitative methodology. Following Hoogeveen (2018), who found strong correlation between the quality of a country's social contract and its statistical capacity, Cloutier proposes characterization of the social contract across three interconnected elements: the citizen-state bargain, social outcomes, and resilience and dynamism. These elements are then quantified across six dimensions measured by 14 proxy indicators, from which a Social Contract Index is constructed. For the case of sub-Saharan Africa, the indicators are drawn from Freedom House, Afrobarometer, Transparency International, other governance and economic indicator databases.

It is in principle possible to adapt the Cloutier framework to constructing a Social Contract of Research and Innovation Index (SCRII), with parallels to other composite indices such as the UNDP Technology Achievement Index, the Global Innovation Index, and the ArCo index (Archibugi and Coco, 2005). This would require a research project in its own right; the best that may be done here is to sketch out some of the main features that would underpin such an Index, and then to apply these to the case in hand.

Corresponding high-level elements for a contract with research and innovation might then be (i) the citizen-state bargain, (ii) research outcomes, (iii) resilience and dynamism.

The appropriate dimensions for measurement are drawn from the innovation systems approach that has gained a strong foothold in Africa through exchanges between Africa's governments, the United Nations family of organizations, the Development Banks, the European Commission, and development agencies. Indeed, Frascati or Oslo-type surveys have been conducted in more than 30 African states. Indicators for human resources, expenditure (infrastructure), and innovation activities are available from the UNESCO Institute for Statistics, Global Innovation Index, World Bank Indicators, the WIPO, and the Global Competitiveness Index.

Six dimensions to measure the functionality of national innovation systems are proposed:

- Framework conditions including STI policy, finance, incentives, and stability [Martin categorization; GERD: GDP; GERD; BERD].^3^
- Human resources [Researcher FTE/million; Researcher gender ratio; higher education and TVET participation rates].^4^
- Knowledge infrastructure [higher education and PRO diversity].
- Knowledge exchange including openness and linkages [international staff and students; intermediaries; staff mobility; co-authorship; foreign sources of funds; participation in Big Science; professional academies of science].
- Policy learning including measurement, monitoring, and evaluation [regularity of monitoring and evaluation; availability of annual and performance reports].
- Outcome measures [innovation surveys; registration of intellectual property].

These dimensions reflect the emphases found in for example, the OECD Innovation Imperative (OECD, 2015) and European Innovation Scoreboard (EU, 2020).

The main interest of this contribution is the social contract, that receives direct expression *via* the framework conditions that includes STI policy instruments. Reference is therefore made to the Martin categorization (Table 1), augmented by application of Theory of Change that elicits the stated or implied assumptions of STI policy instruments by examining the way that their objectives drive attributable outputs. Theory of Change is a variant on Logical Framework and Results-based Management approaches (Bester, 2012). It advocates that policy should be conceptualized from intended outcomes down to activities and inputs that are deployed to attain these outcomes. The tool has similarities to environmental back-casting, save that back-casting works from an uncertain future to the present, whereas policy is expected to be predictive (Dreborg, 1996). Theory of Change has grown in prominence in the assessment of development programmes and speaks to the causal linkages generating outcomes from intermediate inputs (Weiss, 1998; Vogel, 2012).

Following Hoogeveen's criterion, the existence of, and robustness of country statistical capability is an important element of the political social contract. The availability of STI indicators is in part a measure of the social contract for research and innovation.

The six dimensions, enhanced with the Martin categorization and Theory of Change are used to characterize STI policy and performance, and where possible to draw out the nature of the social contract. Five country case studies serve to illustrate the approach. The cases have been selected based on geographic diversity, the availability of current STI policy statements, and cover West, Central, East and South Africa.

This study is exploratory, and is intended to open a new field and mode of inquiry. The proposed methodology requires further development and testing. Obvious limitations arise from the paucity of up to date policy STI statements, the varying character of such statements, the absence of measurable objectives therein, and a general lack of STI indicators.

## Continental and Country Perspectives

The next two sub-sections analyze the origins and nature of African continental and then country science/research and innovation policy. The first sub-section covers the evolution of science policy through 40 years of the Organization for African Unity (OAU), that was followed by the African Union. The focus of the analysis is to determine the existence and character of the social contract.

### From Organization of African Unity to the African Union

Many of the newly independent states of Africa inherited modest science systems with research institutions that supported commodity and crop exports, and the necessities of public health. These might be termed bio-medical systems that included a national university, a few secondary schools, subsidiaries of the premier scientific institutions of the metropoles, and commodity-based research organizations that were funded *via* producer levies. Examples are the global network of Pasteur Institutes, and the tea, fisheries, and cocoa research institutes of Kenya, Nyasaland, and the Gold Coast. The British bequeathed the Medical Research Council of The Gambia, and numerous animal and plant health organizations. Not to be outdone, the US military set up an outpost of the United States Army Medical Research Directorate in Nairobi, Kenya. Unfortunately, distracted local leadership, instability, proxy wars, the donor focus on basic education at the expense of higher education, structural adjustment, and paucity of own funds, saw many of these institutions all but collapse during the “lost decades” after 1970 (Easterly, 2001).

This disclaimer noted, a continental policy perspective must of necessity commence with the Organization of African Unity (OAU) *Lagos Plan of Action 1980–2000 for the Economic Development of Africa* that devoted no less than a quarter of its text to matters scientific and technological. Paragraph §65 proclaimed a vision for economic and social development guided by an implicit social contract that linked industrial, trade and science policy:

In formulating their industrial development strategy African countries should bear in mind the need to select suitable technology which will also be socially suitable, compatible with resource endowment, and increasingly to reduce Africa's present overdependence on the developed countries for technology.

The Plan then proceeded to urge “… explicit national science and technology policy which translates the national policy for socio-economic development into technological lines of action, and indigenous inputs (§128).” Moreover, “research undertaken at universities and other institutions (should be) geared to development needs (§150).” Member States were tasked with “gradually reaching the target of mobilizing, at the domestic level, 1 per cent of their GDP for the development of their scientific and technological capabilities (§52).” This would be funded from own sources, supplemented by the “allocation of a certain percentage of taxes derived from the consumption of imported items to the R&D activities aimed at producing their equivalents locally and for using local resources,” levies on all enterprises, insistence that foreign firms conduct their R&D locally, failing which they would be subject to levies payable to a national R&D fund.

The Plan exhorts attention to the six dimensions especially through government intervention (Martin 2), and by addressing demand pull (Martin 6). It exhibits a strong appeal to resource nationalism and overcoming structural dependency and exploitation. The Plan was punitive to foreign direct investment in seeking to raise funds for R&D through additional tax levies. Theory of Change analysis is inapplicable to such a high-level instrument.

At the time of its crafting, STI indicator development across Africa was at its infancy, so that populating the indicators for the six dimensions would have been nigh impossible.

The Lagos Plan Influenced African S&T policy thinking for a generation. The one per cent target for GERD: GDP was never attained, and the rhetoric of S&T for development was beached.

One of the final initiatives of the OAU before its reconstitution as the African Union (AU) was the establishment, under the patronage of the Presidents of Senegal and South Africa of the New Partnership for Africa's Development (NEPAD). NEPAD functionaries duly crafted *Africa's Science and Technology Consolidated Plan of Action* (African Union, 2005). The Plan, colloquially known as “AMCOST” was structured toward capacity building, knowledge production, and technological innovation. AMCOST, in contrast with the Lagos Plan is technocratic, declaring:

Knowledge production is really about the conduct of science—the generation of scientific and technical knowledge about Africa's problems and identification of specific ways to solve the problems. This is what is often referred to as R&D. Technological innovation entails the generation of specific products, processes, and services (idem: 5).

Noting the continuing dependence on foreign funding, and the failure to support science and technology as engines of development, AMCOST emphasized the importance of programmes on the ground, renewal of research institutions, and the founding of flagship projects. The Plan also launched the African STI Indicators Initiative (ASTII), the African Observatory of STI, and the *African Innovation Outlook* series. These would strengthen STI statistical capability, noted earlier as a key component of any social contract. In reality but a handful of African states have been able to generate and maintain STI indicator time series, reasons being a general weakness in statistical capability and limited funding for STI indicator measurement and collation.

AMCOST promotes a return to basics, science for scientists, and development of scientific institutions. AMCOST celebrates excellence, the tacit assumption being that excellent science, for which read R&D, will translate into public good. AMCOST is highly specific on R&D thrusts, but is silent on the link between science policy and industrial policy. AMCOST thereby straddles Martin categories 1, 2, 6, and 14. Theory of Change is implicitly that of a linear model of innovation.

The orientation of AMCOST might be understood because of the overarching power of South Africa's science system on the continent. No other African state compares in terms of depth and breadth of industrial diversification, strength of scientific institutions, or innovation outputs. In the formalism of Dependency Theory, South Africa may be a periphery of the North, but simultaneously acts as a sub-metropole for its South. Postgraduate students flood from Sub-Saharan Africa to South Africa; the country is second to China as a source of foreign direct investment to Africa and her transnational corporations are found across the continent.

It may be observed that the NEPAD headquarters are in Pretoria, South Africa, with the NEPAD Science Secretariat located on the CSIR Campus. The NEPAD, SADC and African Union STI agendas exhibit a strong South African influence (NACI, 2009), as demonstrated in the choice of its four clusters of flagship R&D projects—biodiversity, biotechnology, and indigenous knowledge; energy, water and desertification; material sciences, manufacturing, laser and post-harvest technologies; information and communication technologies and space science and technologies, that are part of the South African public sector research agenda.

Under the leadership of South Africa's former Minister of Foreign Affairs, the African Union then proceeded to develop a 50-year vision, *Agenda 2063: the Africa we want* (African Union, 2013) that envisaged a knowledge society of well-educated citizens, and a skills revolution underpinned by science, technology and innovation. Agenda 2063 places stronger emphasis on technology, rather than science or innovation, and espouses self-reliance and sovereignty. This advocacy may be read as a call to reduce dependency relationships, including foreign aid and enclave foreign direct investment.

*Agenda 206*3 subscribes to the triple canon of the rule of law, capable, and accountable government. In addition, “economies should be structurally transformed to create shared growth, decent jobs and economic opportunities for all (idem: 3).” This canon mirrors that of the Washington Consensus, but stops short of explicating the nature of what structural transformation might entail. As to sovereignty and self-reliance, dependency remains hard-wired into the system, as the core budget of the African Union Commission derives from international sources, rather than from Africa itself. Arguably the canon is an encrypted message—“We are a trusted partner that shares your values. We can cooperate. Fund us.”

In contrast to the technology thrust of *Agenda 2063*, the subsequent *Science, Technology and Innovation Strategy for Africa (STISA) 2024, on the Wings of Innovation*, gives more attention to innovation, identifying the need for building research infrastructure, enhancing professional and technical competencies, promoting entrepreneurship and innovation, and the provision of an enabling environment for STI. The drafting of *STISA 2024* was co-chaired by Prof. Calestous Juma, of Harvard Kennedy School and Prof. Ismail Serageldin Director of Bibliotheca Alexandrina, and re-iterated the call that “… each Member State is encouraged to take concrete actions to allocate at least 1% of GDP to R&D … (African Union, 2014a: 41).” *STISA 2024* notes the importance of an indicator framework, and a monitoring and evaluation plan “… which outlines the problem, major drivers of performance with regard to effectiveness and efficiency, and a Logical Framework which links goals, objectives and actions. Links with the continental process for harmonization of Statistics in Africa will also be established (idem: 50).”

*STISA 2024* is firmly technocratic in nature, speaking to functionality dimensions 1 through 6, especially the latter. The Martin categorization points to early stage STI policy as well as more contemporary formulations. The emphasis on MEL and Logical Framework is noteworthy. The implied social contract remains that of trusting the scientist, a celebration of the Republic of Science.

The continental average of GERD: GDP stands short of 0.4% (UIS, 2021). The low level of GERD: GDP is an indicator of input weakness. Output weaknesses are the low levels of registration of intellectual property rights, including plant cultivars. At aggregate level, consistent with country shortcomings, the continental STI indicator system remains underdeveloped.

### Country Level STI Policy

Compiling an inventory of science policy statements is a research task in its own right. CREST (2014) identified 17 Sub-Saharan Africa (SSA) countries that had STI policies or strategies in place. More recently African Union (2019) reported that 25 African countries had developed an STI policy statement.

To this number may be added the six Maghreb states, and Egypt, all of whom were early formulators of STI policy, plus Angola, Mauritius, Madagascar and Sudan,^5^ and more recently Sierra Leone, The Gambia, and Lesotho, giving a total of at least 31 states with STI policy in place, or close to adoption. This list includes 15 of the Least Developed Countries.

Detailed analysis of the above array of policy statements is beyond the scope of this contribution, so analysis must largely draw on secondary sources. These include the summary report of the Science Granting Councils (CREST, 2014), and the counterpart study of the political economy influence on STI of SSA countries (Chataway et al., 2017). To these may be added the UNESCO GO-SPIN studies of Botswana, Malawi, Mozambique, Rwanda, and Zimbabwe. The Southern African Innovation Support programme provided overviews of Botswana, Namibia, Zambia, and Mozambique (SAIS, 2014). For North Africa there is corresponding information for Morocco (ESCWA, 2016a), Tunisia (ANIS, 2013; ESCWA 2016b), Egypt (ARST, 2015), Sudan (ESCWA, 2016c), and for the Arab World in general ESCWA (2017).

CREST (2014) found that the installation of a centralized government department responsible for STI policy and governance generally took place in the second generation after independence, and in one case had yet to emerge. Science policy therefore had little time to evolve and adapt. In such an environment, it is unsurprising that science granting councils have low status. Indeed, the experience of these bodies serves as a proxy for the larger issues that hold back STI, namely shortfalls in funding, incentives, governance structures, priority setting, foreign donor influence, losses of the highly skilled through brain drain, and coordination failures.

The situation in North Africa is somewhat different—longer periods since independence and strong linkages with the neighboring French system. Morocco has made considerable gains over 2012–2016, with the adoption of the Moroccan Innovation Initiative and Horizon 2025; Egypt has revised earlier formulations through the National Strategy for Science, Technology and Innovation (2015–2030), alongside the Technology Innovation and Entrepreneurship Strategy and the National ICT Strategy and the impactful EU-Egypt RDI program. Tunisia has introduced a wide range of innovation policy instruments across government, including governance structures, incentives, and intermediaries, though ESCWA (2016b) finds that the absence of an overarching strategy may be a brake on achievement. Sudan has also introduced a new STI policy (ESCWA, 2016c). For its part Algeria is seeking to move away from dependence on hydrocarbon exports, with its strategy for research running from 2009 through 2017. In summary ESCWA (2017:2) notes that “institutional frameworks remain largely insufficient to foster and regulate innovation, and market sophistication (financing) is weak, although infrastructure meets global averages.”

Iizuka et al. (2015) assessed the STI policy of 14 East, Central, and Southern African states against six priorities. For the 10 countries for which data was available they scored research capacity (45%), human resources (45%), researcher networks (30%), ICT (55%), institutional capacity (47%), and linkages with the private sector (22%). The highest scores were recorded for Zimbabwe; the lowest in Namibia and Botswana. Nine of the 10 countries had revised their policies to include an “Innovation” thrust that might explain why ICT tended to score well in most countries.

Of the three African Innovation Outlooks published to date, the second, African Innovation Outlook 2 (African Union, 2014b) provides insight into the fields of interest of the STI policies of 11 sub-Saharan countries, finding these still predominantly bio-medical.

UNECA (2016) reviewed the policies of 15 states, Angola, Botswana, Ethiopia, Gambia, Ghana, Kenya, Lesotho, Malawi, Nigeria, Rwanda, South Africa, Tanzania, Uganda, Zambia, Zimbabwe, concluding that while these policies had led to organizational changes and the introduction of legislation, they showed “cross-cutting weakness, that is, the inability to estimate the cost of implementation. This weakness may explain the poor outcomes (idem: 86).”

For its part the 2017 Africa Capacity Report on STI (ACBF, 2017) avers that two-thirds of African countries have STI policies and strategies, but their capacity to implement them remains very low. CREST (2014: 20) supports the position: “… a commitment to a science policy or Ministry of Science and Technology is not sufficient if it is not accompanied by a significant investment in R&D in a country.”

The most recent continental assessment is the Ridley-Offiong review Africa's STI Implementation Report 2014–2019. The authors note that progress is being made in implementing STI, in that at aggregate level countries “perform equally as well as their counterpart countries with similar GDP levels in other regions,” (even as) “… challenges facing the weakest countries of the region in STI are exemplified by the fact that many countries are unable to even provide adequate data for assessment (African Union, 2019: 9).”

As to linkages, the very concept of an innovation system is predicated on the existence of productive linkages among the main actors—higher education, business and government, with the Triple Helix formulation (Etzkowitz and Leydesdorff, 1998, 2000) giving stronger attention to the role of universities.

The perceived dysfunctionality of emerging economy innovation systems led Albuquerque, 2003 to use STI indicators to catalog Argentina, South Africa, Brazil, and India as “immature” innovation systems. Soete and Freeman (2007) are more discrete, referring to the “disarticulated” innovation systems prevalent in developing countries whose universities behave much as the inward-looking ivory towers of the pre-Humboldtian era.

**Figure 1:**
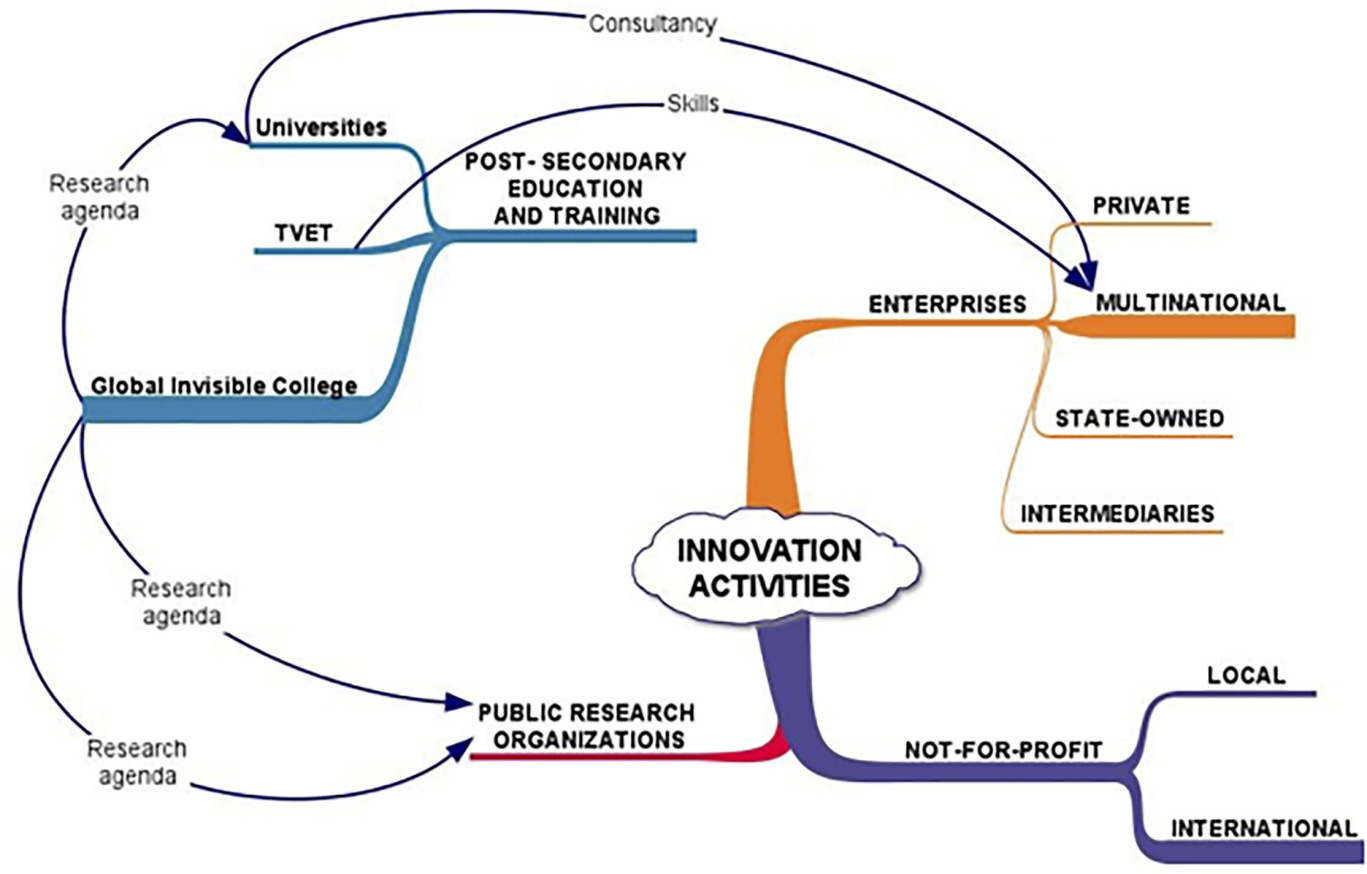
A disarticulated innovation system. Source: Author.

Figure 1 is a schematic of a disarticulated innovation system. The schematic eschews the straight lines and boxes that typify models of innovation systems, alluding to the complex-self organizing, sometimes chaotic nature of these “systems.” It highlights the gaps between the main actors and their limited engagement in innovation activities. The schematic emphasizes the strong links between African universities and the global invisible college of science. Associated co-authorship, affiliation, and mobility may be studied through bibliometric analysis.

In disarticulated innovation systems, the universities have a stronger linkage with the global invisible college of science than to domestic actors. Disarticulation is shown explicitly as the gap between the post-secondary education and training system and the other innovation system actors. Internal flows and linkages include consultancy services, the provision of skills, and the imposition of research agendas that arise through the inability of many countries to provide funds to drive national research agendas (Hanlin et al., 2021).

The international research agenda often takes the form of “big science” as in projects such as the Global Burden of Disease, high-energy physics at CERN, astronomy and astrophysics as in the Planck Telescope, and the massive effort of clinical trials of COVID-19 vaccine candidates.

The fact that African countries participate in such international projects attests to the excellence of her scientists. However, the domestic role of the universities is inadvertently reduced to the provision of the highly skilled for employment in government, the services sector, and industry to some extent. The low barrier of entry permits some graduates to forge ahead as young entrepreneurs who create financial technology start-ups in the services sectors, as are now proliferating in countries such as Nigeria, Tunisia, Kenya, Ghana, and South Africa. A second knowledge flow from the universities takes the form of consultancy services to government, the donor community and industry by university staff with the appropriate skill (and time) to do so.

More recently, Twiringiyimana et al. (2021) have used the Triple Helix and innovation system approaches to identify gaps between governance and policy in SSA countries, and to make suggestions for policy.

None of the above high-level reviews make direct enquiry into the existence of a national level social contract for research and innovation. Following Cloutier, it may be averred that elements of a social contract are found in those country that present a recent STI policy statement coupled with timely and complete STI statistics. To varying extent Algeria, Egypt, Ghana, Kenya, Nigeria, Tunisia, and South Africa meet this test.

In order to probe more deeply for evidence of a social contract, the five country case studies follow.

## Five Case Studies

### Ethiopia

The Ethiopian polity was, and remains troubled, the country having progressed through the overthrow of monarchy, decades of conflict and civil war. The country is landlocked, has wide climatic variation and in principle is able to feed herself. Ethiopia is a *de facto* one-party state that follows a planned economy and is a Least Developed Country, with the goal of achieving middle-income status by 2025.

The National Science Policy and Strategy (NSPS) of 2020 seeks to “build a knowledge-based, technology-driven economy and society through the creation of capability in human resources, infrastructural and technological capability, science education, basic and applied research, and dissemination of knowledge and research outputs as deemed necessary (MSHE, 2020: 30).” The NSPS declares that the Ministry of Science and Higher Education will provide strong advocacy for science, ensure coordination and harmonization of the NSPS and its programmes across research organizations, science-affiliated institutions, professional societies, HEIs, and TVETs' (idem: 33). “Apart from facilitating the development of human capital, high-standard scientific research, scientific discoveries, this Policy and Strategy will enhance the careers of scientists and their competitiveness and afford them visibility globally (idem: 10).”

The Ethiopia policy is in the early stages of Martin's typology. Government STI statistics are collected, but sporadically. The core components of the NSPS read as a generic action list spanning adult education, science promotion, building a science elite, and unspecified industry support. A National Research Council and “science and institution fund” are proposed, without detailed motivation. In effect the NSPS declares a state-led agenda with the Ministry of Science and Higher Education as driver, with the HEIs expected to engage in relevant knowledge production.

Ethiopia's STI indicator system is limited, and does not offer regular measurement of GERD, BERD and Researcher FTE/million. Publication output stands at an annual 44 per million over 2017–2021, with highly cited papers in the health sciences, especially public, environmental and occupational health.

There is little evidence of innovation systems thinking or application of a theory of change. The bargain would appear to be support for science alongside the promotion of the interests of scientists.

### Rwanda

Rwanda is a *de jure* multi-party democracy, but in reality, operates as a one-party developmental state. The country is land locked, and densely populated, with fertile, arable lands and massive agricultural, mineral, and renewable energy potential. The country continues with post-genocide reconstruction, with considerable effort going toward developing knowledge infrastructure.

Research and innovation have been given prominence in Rwanda's Vision 2050 that expresses the goal of attaining middle income status by 2035 and high-income status by 2050. The 2020 Rwanda National Science, Technology, and Innovation Policy (NSTIP) reflects these goals, referencing progress made in the socio-economic domain, and policy learning since the adoption of the 2005 S&T Strategy, including the UNESCO GO-SPIN report of 2015, and the UNCTAD STI policy review of 2017. A National Research and Innovation Fund (scientific research and research-led innovation) and the National Research Fund (startups and tech-enabled SMMEs) were established in 2018.

Even so, STI performance is deemed to be below par, and the NSTIP is intended to “fast-track and strengthen the performance indicators within the National System of Innovation in line with the National Strategy for Transformation” and to plug systemic gaps such as coordination, fragmented initiatives, weak linkages, and sup-optimal resource concentration (idem: 2). The mission is to establish “a vibrant STI environment … to cater for the needs of the productive sector and society” through five objectives, effective governance, increased S&T output, increased R&D and innovation funding, improved S&T capacity and networks, and international collaboration. These objectives read much as a standard set of World Bank/OECD precepts.

To give substance to direction setting, an Annual Joint Planning Session is to identify specific STI activities with government to monitor progress, complemented with a host of supply-side measures—centers of excellence, science parks, incubators, and accelerators, including R&I departments in the private sector and universities, and the commercialization of indigenous knowledge.

This top-down strategy gives attention to emerging technologies irrespective of their immediate relationship with economic development and begs the questions of government span of control and span of influence. The NSTIP is multifarious in intent, declaring mandatory university-industry participation as a requirement for funding, establishment of private R&D funding instruments such as venture capital, crowdfunding, seed funds and donations, incentivization of industry, and a “mechanism to promote research careers across the whole R&D and innovation value chain.” Six priority “sectors” are identified, namely sustainable energy, food security and Modern Agriculture, Life and Health Sciences, Local production, and value addition [not a sector], digital services, resilient environment and climate change, and the implementation section is accompanied by an array of 51 unprioritized key policy actions costed out to 2024. A theory of change is embedded in the 51 unprioritized key policy actions but the steps and stages to be followed cannot be deduced from the listed actions. STI indicator collation is very weak. Publication output stands at an annual 51 per million over 2017–2021, with highly cited papers in the health sciences, especially clinical medicine.

NSTIP may be categorized as a science-push policy that corresponds with the early stages of the Martin typology, with some aspects demonstrating more contemporary approaches. The implied social contract might read as: “government knows what is required and will deliver.”

### Senegal

Senegal is a multi-party democracy whose innovation system hosts a few colonial-era PROs and respected universities that draw in migrant students from Francophone Africa. By design, and with other Francophone states, Senegal maintains strong links with the French science system, with nine of the top 10 foreign institutions for academic collaboration being in France. Publication output stands at an annual 57 per million over 2017–2021, with highly cited papers in the health sciences, especially clinical medicine.

This appraisal recognizes Cissé et al. (2019) who note that despite there being an organization dedicated to higher education, research, and Innovation (*Ministère de l'Enseignement Supérieur, de la Recherche et de l'Innovation*), no specific STI policy instrument is in place, and that this has led to a lack of coordination, and little productive interaction with stakeholders. The proliferation of dedicated funds (Scientific and Technical Research Fund of 1979; Scientific and Technical Publication Fund; National Fund for Agricultural and Agro-Food Research Fund of 1999; Fund for Financing Professional and Technical Training of 2014) and the Presidential Grand Prize for Science and Technology, give the impression that the government has long-recognized the importance of building a scientific elite, further evidence of which is evidenced by the high pay accorded to university staff (idem: 6). Indeed, this favors “research for international academic and scientific excellence rather than endogenous research (idem: 8).” GERD: GDP was estimated at 0.8% that places her among the African leaders. STI indicator collation is institutionalized, but does not produce regular measures of GERD, BERD or Researcher FTE/million.

A recent shift of attention to issues of market access and employment was announced through the Delegation for Rapid Entrepreneurship of 2017, but this has not borne fruit, and the business sector remains a mix of under-financed SMMEs who are locked out by large monopolistic multinational corporations. There is little interaction between PROs and the private sector, so that the system might well be described as disarticulated, and insufficiently focused on applied research and commercialization.

The undocumented social contract takes the form of “science for scientists” and for national prestige, without an explicit theory of change. This places the Senegal policy in Martin's early stages of STI policy.

### Sierra Leone

The fourth case is that of democratic Sierra Leone, also among the Least Developed Countries. Sierra Leone has emerged from decades of misrule, civil war, natural disaster and epidemic, during which there has been considerable loss of life and livelihoods, population displacement, and loss of skills through flight and emigration. The promise of responsible government and economic recovery has spurred some of her diaspora to return and contribute to nation-building.

The government that took office in 2018 created a Directorate for STI (DSTI) under a Chief Innovation Officer in the Presidency. The Directorate is responsible for STI policy, direction setting and coordination, and is also, uniquely, a research and innovation performer. On the policy front the DSTI developed the National Innovation and Digitization Strategy (NIDS) (2019–2029) “that is driven by one core philosophy—Digitization for All: digital identity; digital economy; and digital governance (DSTI, 2018: 6).” This triple thrust entails (i) the assignment of unique identifiers to natural and juristic persons, to institutions and assets, (ii) building digital payment and banking systems and infrastructure, and (iii) secure, seamless interaction between government and citizens.

DSTI is more than a policy formulator; it houses a team of system engineers who are active in designing and rolling out the necessary applications to attain the strategic goals. The funding for these activities is sourced from government and international donors.

The strategy assumes that good governance, the rule of law, and evidence-based decision making will apply. By design the strategy is technocratic and narrow, relying on digitization roll-out to bring about social change. A limiting factor is the robustness of the national communications backbone as well as limited access to smartphones and broadband. Another limiting factor, far from unique to Sierra Leone, is the extent to which government departments will positively engage with the DSTI agenda. Attaining open, efficient government is a common mantra whose fulfillment requires numerous supportive actions.

Each project requires its own milestone plan that expresses the stepwise theory of change. NIDS does not provide such and has a critical dependence on the vision and capabilities of the Chief Innovation Officer, who is also the Minister of Basic and Secondary Education.

The Martin categorization suggests that the National Innovation and Digitization Strategy may be described as a demand-led, mission response that advances mechanisms to achieve social inclusion. The science system is small, with publication output over 2017–2021 of 1,170 records on the Web of Science (29 per million population) and 70% in the health sciences. The unique burden of disease is such that many health sciences papers are highly cited. STI indicator system is very weak.

Digitization for all bears the hallmarks of a technological mission, driven to address evident shortcomings and to achieve well-defined, short, and medium-term goals. The social contract is explicit: investment in digital solutions (applied science) will result in a connected inclusive society, this in a polity recovering from three lost decades.

### South Africa

South Africa is a middle-income country with the highest level of industrial diversification in Africa, whose history includes *apartheid*, atomic weapons, and the world's first human heart transplant. The economy rests on its minerals-energy complex that is both resource curse, and the support-base of a now extensive welfare state. The polity grapples with her own version of the middle-income trap (Luiz, 2016). South Africa's scientific institutions have a two-century history, and a few her universities are among the world's top 500. Her scientific outputs tally for pole position with Egypt, with her share of the most highly cited publications in continental pole position. The diversified science system boasts pockets of excellence, but lacks critical mass as the stock of researchers has not grown apace.

Mission-oriented scientific research began in the 1920s through the then Department of Industrial Research, indicating a long history of attention to science policy. The earliest S&T policy dates from the 1960s when the country began its slide into authoritarianism, civil war, and militarization. Pre-1994, the CSIR, still the largest PRO in Africa, played the dual role of S&T performer and the seat for S&T policy advice to government.

The introduction of constitutional democracy in 1994 called for a re-appraisal of the role of S&T in development. This was expressed in a White Paper (DACST, 1996) that was then followed by the National R&D Strategy (DST, 2002). The White Paper was a mechanism to introduce new thinking and new institutions, such as the National Advisory Council on Innovation, and the Innovation Fund. New thinking included the explicit declaration that the innovations systems approach was to be followed, with an innovation system understood to be “a set of functioning institutions, organizations and policies which interact constructively in the pursuit of a common set of social and economic goals and objectives (DACST, 1996: 20).” Furthermore, “the promotion of research, both applied and basic, in the natural sciences and in the social sciences, is crucial to innovation and hence to both social and economic development (idem: 21).” While applied science would address shortcomings in the social domain, basic research would be incentivized, to support physics and astronomy flagship projects. “Not to offer them would be to take a negative view of our future—the view that we are a second-class nation, chained forever to the treadmill of feeding and clothing ourselves (idem: 16).” The White Paper expresses a mixed social contract: support for basic research, driving flagship projects, and meeting developmental needs, the new institutions were established, and the document reads as both a vision statement and action plan.

**Figure 2:**
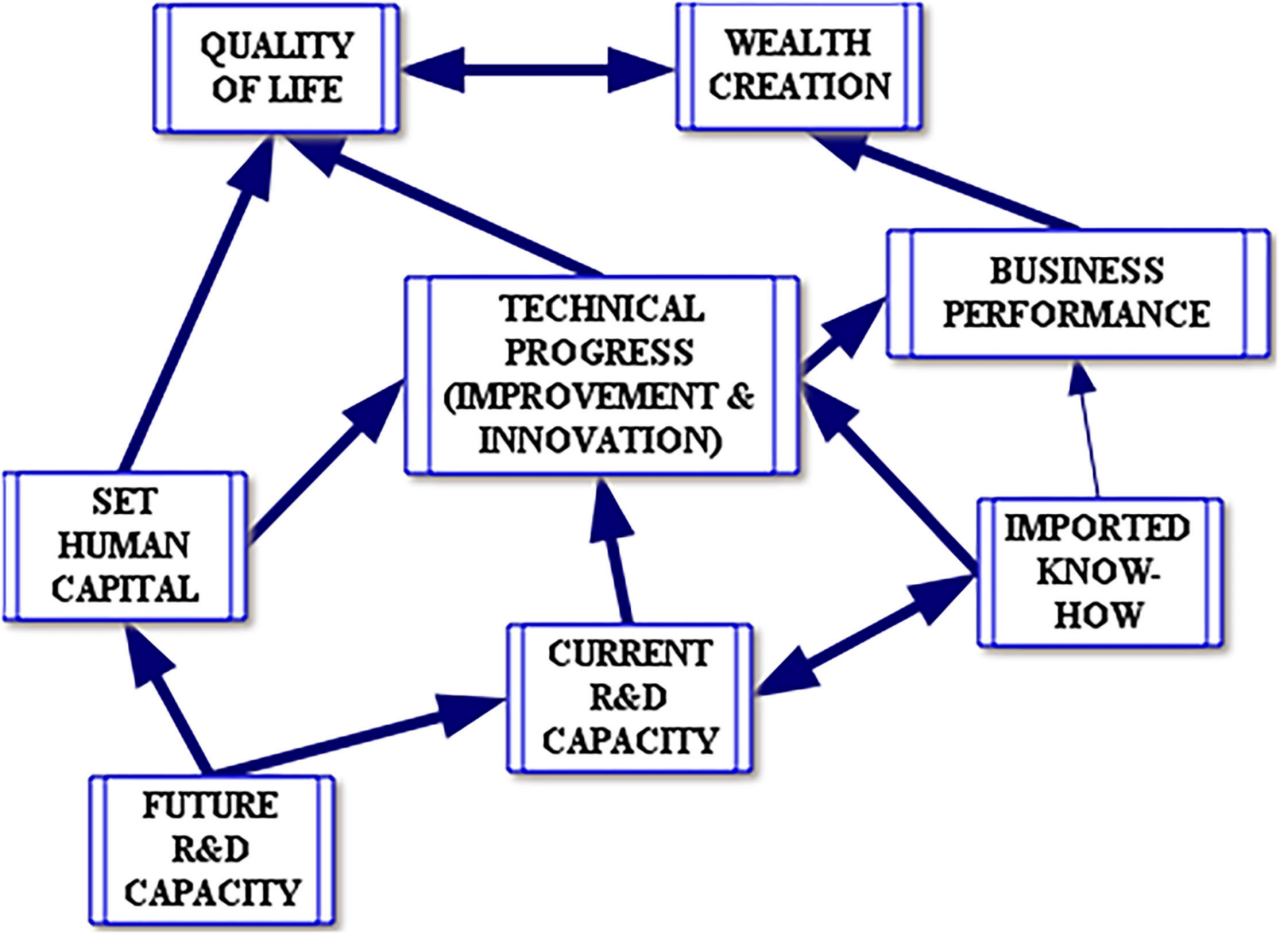
From capacity to outcomes: how R&D impacts economic growth and quality of life.

In contrast, the 2002 R&D Strategy reads more as a vision statement than a strategy. In support of this contention, reference is made to the organizing schema of DST (2002) (Figure 2), that depicts how support for R&D will lead to economic and social change. In effect the schematic is a theory of change. “If then, such and such will follow.” The schematic had a powerful effect on South African STI policy from 2002 through to 2019 (DST, 2002), appearing in policy statements of the Department of Science and Technology, the National Advisory Council on Innovation, and the Technology Innovation Agency that took office after 2009.

The schematic, with its implied theory of change was developed using the decision-making application Think Tools AG.^6^ This explicitly linear model of change places R&D as the font of new knowledge that will percolate upwards to improve the quality of life and economic growth: “Modern economies require all these elements to be present and growing. This framework is a representation of the National System of Innovation (NSI). The two major outcomes required from R&D and innovation are increased wealth and quality of life. There is incontestable evidence that this process requires ongoing public sector investment (DST, 2002: 28).”

However, the Strategy was not accompanied by a plan, nor specific auditable objectives, save for targeting GERD: GDP to reach 1% by 2008, and for government spending on R&D (GOVERD) to double in nominal terms. The 1% target was not attained, but GOVERD doubled. However, the long-term trend is that the share of business expenditure on R&D (BERD) has declined, with a shift toward basic research that now accounts for 32% of GERD. Publication output stands at an annual 472 per million over 2017–2021, with highly cited papers in the health sciences, especially clinical medicine.

South Africa serves as the African exemplar of a well-supported STI indicator system. Indeed, her overall national statistical system continues to enjoy wide respect.

Neither the quality of life, nor wealth creation have risen significantly in the period post 1994, and holding STI policy accountable for this would be misplaced. Flagship projects—the Pebble Bed Modular (nuclear) Reactor, the Joule electric car, and the 64-dish MeerKat radio telescope array—have promoted the image of research and innovation but have not gained industrial traction.

Kahn (2019) described the underlying social contract as one in which science marched on two legs: one leg representing the Republic of Science, the other representing state-driven flagships.

The Martin categorization sets the R&D Strategy as spanning stages one through ten. This suggests a linear model, science-push approach that has been disseminated into STI policy circles nationally and further afield.

## Discussion

The above cases illustrate policy in action, and sometimes inaction. The selection may be criticized as limited in number, but it does suggest that STI policy in Africa is constrained by science push, and linear model thinking.

The selected countries, South Africa included, are all trapped in dependency relationships with the advanced economies, on whom they rely for technology imports. Chang (2007) avers that this dependency is exacerbated by the various barriers to market entry that the advanced economies have erected to exclude competition from newcomers. In his words, they raise the very ladder that they had climbed to reach their now high-income status. This poses severe difficulties for the trade, industrial, and innovation policies of the emerging economies and LDCs.

Lundvall (2007) seeks to understand the limitations of the innovation systems approach to developing countries, noting the lack of scientometric data for policy makers and the barriers facing entrepreneurs. Lundvall avers that in developing countries the state must play a direct role to play in “the mobilization of autonomous forces outside the market to create economic development (idem: 117)” that would support entrepreneurs who identify “slack” in the market, *a la* Hirschman. As Martin (2015: 28) notes (in the post-industrial world), “the focus of our empirical studies has not always kept pace with a fast-changing world, in particular the shift from manufacturing to services and the growing need for sustainability and enhanced wellbeing rather than just economic growth.” Economic dependency may be breaking. One must look for slack, harness expertise, and drive forward. Another example of such perseverance is Lesotho's Matekane Group of Companies^7^ that serves as an exemplar of innovation under severe constraints.

While much is made of the to be 4th Industrial Revolution, the fact is that the ICT technologies, unlike those of the machine revolutions, offer a soft, knowledge intensive route to participation and innovation. The case of the Sierra Leone DSTI stands out in this regard. So too the fintech startups and “unicorns” with African roots.^8^ San-Francisco-based Flutterwave was founded by Nigerians Agboola and Aboyeji; Interswitch, is headquartered in Lagos, and owns Verve that accounts for 80% of Nigeria's payment cards. Egyptian electronic payment company Fawry has 30 million subscribers, while Nigerian e-commerce platform Jumia, originally co-funded by South Africa's MTN, had by 2016 become the first African unicorn. These developments square with the earlier findings of the Danish DISKO project that “business services have become a kind of strategic sector playing a role like the role played historically by the sector producing machinery in the industrial economy (idem: 105).”

System and software engineering has a relatively low barrier of entry: logical thinking, appreciation of a market gap, programming and algorithm skills, a laptop and broadband connection. These enablers relegate underdevelopment as an attitude of mind.

This is not to suggest that the playing field has been fully leveled. Some partly open goals beckon, but getting within striking range remains a challenge.

The task facing the national STI policy formulator is daunting. Talking up the importance of investment for STI against the needs of industrial policy; balancing competing interest groups, including academia, other government departments, public research organizations, and civil society interests, lobbyers, the donor community, and high-profile scientists; demonstrating value for money.

To this add the warnings of the original Sussex Manifesto: how to counter “internal brain drain” compounded by the understandable ambition of foreign-trained scientists who return home with deep knowledge in frontier science that may not have an easy fit at home.

This calls for a careful balancing act that avoids the pitfall of offering all things to all people. How to focus on the important; how to marshal the resources; how to deliver? Traditional matters of any strategy, but always pertinent.

STI policy has served to build local scientific elites, by promoting the excitement of scientific discovery, and the ability of Africans to participate as equals. Celebrating scientific achievement is a product of STI policy and promotion, and the proliferation of Academies of Science testifies to this. On the other hand, the valorization of research is limited by disarticulation between industrial and innovation policy, and the gap between research institutions, industry, and society.

As to Africa's research and innovation policy and the COVID-19 Pandemic, long experience in dealing with infectious diseases, strong, purposive engagement to meet the WHO International Health Regulations and, to undergo the associated Joint External Evaluation is an untapped resource.

Bibliometrics shows that Africa has much to contribute in terms of understanding disease etiology and epidemiology, and the treatment infectious diseases, though the track record of developing cures lags. The detection of the SARS COV-2 Beta and Omicron variants are cases in point. South Africa detected these, but paid a heavy price as the messenger, being isolated from the world even as Omicron undetected. Coupled with the high cost of vaccines, the notion of “vaccine apartheid” took hold.

These observations imply that the implicit social contract continues to embraces bio-medical research, even as this is pulled by Western sources of funds and research centers. Path dependence is also evident in science systems.

This contribution began with an admittedly Eurocentric precis of the evolution of the social contract for science, from Bernal's instrumentalism through to Schot and Steinmuller with their call to move beyond what might be labeled Innovation Policy 3.

African science policy is loosely informed by both. At best the latter receives lip service since innovation ecosystems are so constrained.

R&D-led innovation would appear to be an unrealistic goal, consistent with Dependency Theory that suggests that commodity producers will find themselves restricted in their quest for industrial diversification, let alone innovation. Indeed, structural economic weakness continues across Africa. The continental policies constitute non-binding advocacy to promote STI for development. The five cases studies confirm the research bias toward the health sciences noted in African Union (2014b) and imply that the implicit social contract embraces health science research, even as this is pulled by Western sources of funds and research centers. A broader social contract for research and innovation is largely missing in action.

## Data Availability Statement

Publicly available datasets were analyzed in this study. This data can be found at: www.uis.stat.

## Author Contributions

The author confirms being the sole contributor of this work and has approved it for publication.

## Conflict of Interest

The author declares that the research was conducted in the absence of any commercial or financial relationships that could be construed as a potential conflict of interest.

## Publisher's Note

All claims expressed in this article are solely those of the authors and do not necessarily represent those of their affiliated organizations, or those of the publisher, the editors and the reviewers. Any product that may be evaluated in this article, or claim that may be made by its manufacturer, is not guaranteed or endorsed by the publisher.

## Footnotes

^1^It is surprising that innovation studies are a post-War phenomenon. Much could be learnt from studying technological learning during the inter-War period, that saw Japan stride forward with impressive vigor.

^2^Prof. Mazzucato serves on South Africa's Presidential Economic Advisory Council, is a member of the Scottish Government's Council of Economic Advisers, and was a member of the UK Labor Party's Council of Economic Advisers from 2015 to 2016. https://www.thepresidency.gov.za/press-statements/president-participate-national-school-government-master-class-presented-prof-mariana-mazzucato.

^3^GERD, Gross expenditure on R&D; BERD, Business expenditure on R&D.

^4^FTE, Full-time equivalent; TVET, Technical Vocational Education and Training; PRO, public research organization.

^5^ http://www.unesco.org/new/en/member-states/singleview/news/revised_policy_may_herald_new_dawn_for_sudanese_science/

^6^ https://www.spiegel.de/politik/geist-aus-der-maschine-a-b2549934-0002-0001-0000-000016525391?context=issue

^7^ www.mgc.co.ls

^8^ https://peopleofcolorintech.com/founders/africas-giants-the-tech-unicorns-ready-to-make-waves/
